# Study on Particulate Structure Characteristics of Diesel Engines Fueled with a Methanol/Biodiesel Blend

**DOI:** 10.1021/acsomega.0c04371

**Published:** 2021-02-23

**Authors:** Ruina Li, Zhong Wang, Chunyi Tang, Xin Meng

**Affiliations:** † School of Automotive and Traffic Engineer, Jiangsu University, Zhenjiang 212013, China; ‡ School of Mechanical and Electrical Engineering, Xuzhou University of Technology, Xuzhou 221008, China

## Abstract

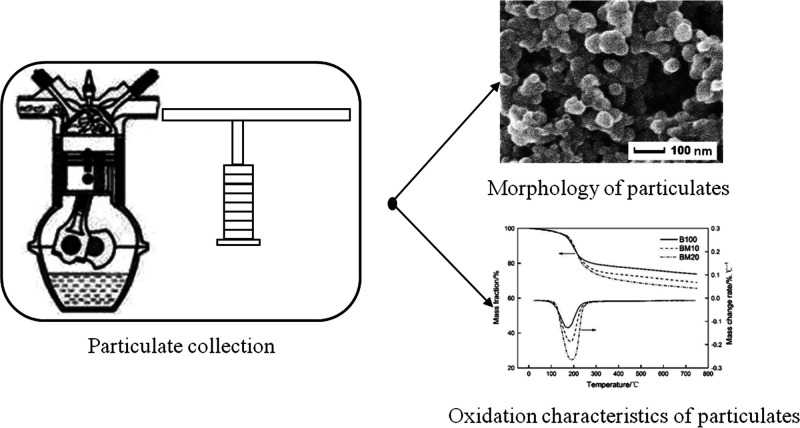

Methanol and biodiesel are both alternative fuels of diesel engines. In order to study the effects of methanol on the microstructure of particulates produced from the diesel engine fueled with a methanol/biodiesel blend, the methanol/biodiesel blend fuels with 0, 10, and 20% methanol were prepared (named B100, BM10, and BM20, respectively). SEM and TG experiments have been carried out, and the structural and oxidative characteristics of particulates for the methanol/biodiesel blend were investigated. The results showed that the average diameters of B100, BM10, and BM20 particulates were 35, 32.6, and 31.2 nm, respectively. With the increase of methanol blending ratio, the H_2_O and SOF (soluble organic fraction) contents were increased and the soot content in particulates was reduced slightly. In addition, the activation energy of the particulate pyrolysis reaction was reduced with the increase of methanol mixing ratio, and the oxidative reaction of particulates was easier to carry out.

## 1 Introduction

Diesel engines are widely used in automobiles, agricultural machinery, construction machinery, ships, railway locomotives, and other ancillary machinery. Soot, NOx, and sulfide generated from diesel engine exhaust are the main causes of haze.^1^ Government agencies have been introducing strict PM (particulate matter) regulations because PM has been classified as “been classified as rict by the international agency for research on cancer since 2012.^2^*Limits and measurement methods for emissions from light-duty vehicles (China 6)* puts forward the limits of particulate quality and limits of the particulate number. Thus, the study on PM can offer information to establish stringent fuel policies. In addition, it can provide knowledge about the impact of alternative fuels on aftertreatment devices (such as diesel particulate filter, DPF).

Since the fossil fuel shortage and global environmental issues have attracted wide attention, many research studies on using alternative biofuels from renewable resources in transportation applications have been carried out. Oxygen-containing fuels, as the clean renewable energy fuels for diesel engines, can improve the combustion process of diesel engines. Alcohols and biodiesel are oxygen-containing fuels and are suitable for use in diesel engines and have been extensively accepted as partial diesel fuel substitutes.^3−5^ Methanol and biodiesel have a wide range of sources and contain 50 and 11% oxygen, respectively. Methanol and biodiesel have good mutual solubility and are easy to prepare blends. Thus, methanol and biodiesel have broad application prospects. Harish’s study revealed that, using methanol in diesel engines increased the combustion duration and cylinder pressure with reduced NOx, PM, and smoke emissions due to reduced ignition delay and higher latent heat evaporation.^6^ As the physical and chemical properties of methanol and diesel were quite different, adding methanol to biodiesel had a great impact on the physical and chemical properties of blended fuel. For example, the latent heat of methanol vaporization was higher than that of biodiesel. When mixed with biodiesel, the combustion temperature in the diesel engine cylinder can be lower, and soot and NOx emissions were less than that of diesel at all loads.^7^ Žaglinskis’s study showed a similar result, which was when 10% methanol was added to biodiesel, the maximum reduction of CO was 13% and the maximum reduction of soot was 45% in diesel engines.^8^

Soot is the main component of particulates. The addition of methanol in biodiesel will influence the combustion process and particulate formation; thus, the microstructures of particulates will be different from those of biodiesel. Because of the low calorific value of methanol compared with diesel, the combustion in the cylinder was reduced, and the cylinder pressure, pressure rise rate, heat release rate, and temperature of the engine fueled with the methanol were lower than those of the diesel.^9^ Tsolakis studied the particulate morphology characteristics of diesel engines fueled with biodiesel. The result showed that the particulates were mainly formed by coagulation and aggregation of spherical basic particles. The diesel particulates were mostly in the form of chains with a loose structure, and the biodiesel particulates were mainly in the form of grapes or clusters with compact arrangement.^10^ The change of fuel characteristics will also affect the mechanical characteristics of particulates. Liu’s study showed that with the increase of methanol content in biodiesel, the attraction force, the cohesive force, the adhesion energy, and Young’s modulus of the methanol/biodiesel particulates were all reduced significantly.^11^

In order to reduce PM emissions of diesel engines, DPFs have become an effective technical measure. Along with the time, the PM adsorbed in the DPF will cause problems such as blockage of the DPF, etc. Performance of diesel engines will be reduced. This involves the problem of the DPF regenerated. Thus, the status characteristics and oxidation reactivity of PM need to be studied.^12^ The composition and microstructure of the particulates have a great influence on the oxidation reactivity of particulates and regeneration efficiency of the DPF. The fuel characteristics and the mixing ratio of multicomponent fuels affect the composition of particulates. Agudelo’s study on crude palm oil, crude jatropha oil, and commercial diesel showed that palm oil presented the smallest primary particulate diameter and exhibited the highest concentration of aliphatics by mass. At the same time, the change of particulate structure also affected the oxidation characteristics of particulates. Thus, the soot of the jatropha oil presented the highest active surface area.^13^ As an important part of diesel engine particulates, SOF (soluble organic fraction) mainly comes from unburned fuel, unburned lubricating oil, and combustion intermediates, and SOF will be adsorbed on the surface of the particulates during the combustion process. The influence of SOF on the oxidation characteristics of particulates cannot be ignored. Zhu’s study on SME50 (soybean oil methyl ester), RME50 (rapeseed oil methyl ester), and PME50 (palm oil methyl ester) showed that the overall sequence of SOF was PME50 > SME50 > RME50, and the SOF fraction in particulates was linear with the saturate fraction of fuel at all test modes.^14^ Yezerets’s study on the factors influencing the oxidation characteristics of diesel combustion particulates showed that adsorbed hydrocarbons provided limited contribution to the overall reactivity of the un-pretreated particulate matter. The first few percent of carbon in particulate matter was oxidized at an anomalously high rate. Further oxidation occurred at a lower rate with rather uniform kinetic characteristics.^15^ Above all, the diesel engine fueled with methanol has a great impact on the composition, structure, and oxidation characteristics of particulate matter. However, the existing research on the characteristics of particulate matter produced by methanol combustion, especially the characteristics of particulate matter generated by methanol biodiesel blended fuel, is less, so it is necessary to carry out further research.

To study the effects of the methanol/biodiesel blend on the particulate microstructure, the SEM and TG experiments have been carried out. The structural and oxidative characteristics of particulates for the methanol/biodiesel blend were investigated, and the average size and oxidation kinetics parameters were analyzed.

## 2 Materials and Methods

### 2.1 Materials

The biodiesel and methanol were provided by a local company in China, and the biodiesel was prepared by esterification of waste oil. The cetane number of methanol was 5, which was much lower than that of diesel. Due to the poor self-ignition characteristics of methanol, the blending proportion of methanol used in diesel engines was less than 30%. In this study, four kinds of blends were prepared: 10% (wt) methanol blending with 90% (wt) biodiesel (named BM10), 20% (wt) methanol blending with 80% (wt) biodiesel (named BM20), and biodiesel was named B100. The test equipment and standard of density and viscosity are listed in Table 1. The cetane number, oxygen mass fraction, and low heat value were calculated according to the Kay mixing law.^16^ The physical and chemical properties of the biodiesel, methanol, and methanol/biodiesel blend are listed in Table 2. It can be seen that methanol had low cetane number, density, dynamic viscosity, and low heat value but high oxygen content. With the increasing addition of methanol, the oxygen content of the blend was increased; however, the cetane number, density, dynamic viscosity, and low heat value of the blend was reduced.

**Table 1:** Test Equipment and Standard Properties of Test Fuels

parameters	test equipment	test standard
density	SYD-1884 densimeter	ASTM D891-09
dynamic viscosity	NDJ-5S digital rotary viscometer	ASTM D2196-2010

**Table 2:** Physical and Chemical Properties of Test Fuels^a^

parameters	cetane number	density (g·mL^–1^)	oxygen mass fraction (%)	dynamic viscosity (mm^2^·s^–1^)	low heat value (MJ·kg^–1^)
methanol	5	0.796	50	0.61	20.2
B100	55	0.88	11.2	6.2	37.3
BM10	45	0.871	15.1	4.4	35.6
BM20	41	0.863	19.0	3.9	33.9

a Note: density and dynamic viscosity tested at 20 °C.

### 2.2 Methods

In order to study the particulate structure characteristics of the methanol/biodiesel blend, the diesel engine bench test was carried out and the particulates of the methanol/biodiesel blend were collected using a micro-orifice uniform deposition impactor (MOUDI). Then, the SEM test was conducted to observe the micromorphology of particulates collected. Through the thermogravimetric test, the oxidative characteristics of the particulates can be investigated.

#### 2.2.1 Particulate Collection

**Figure 1:**
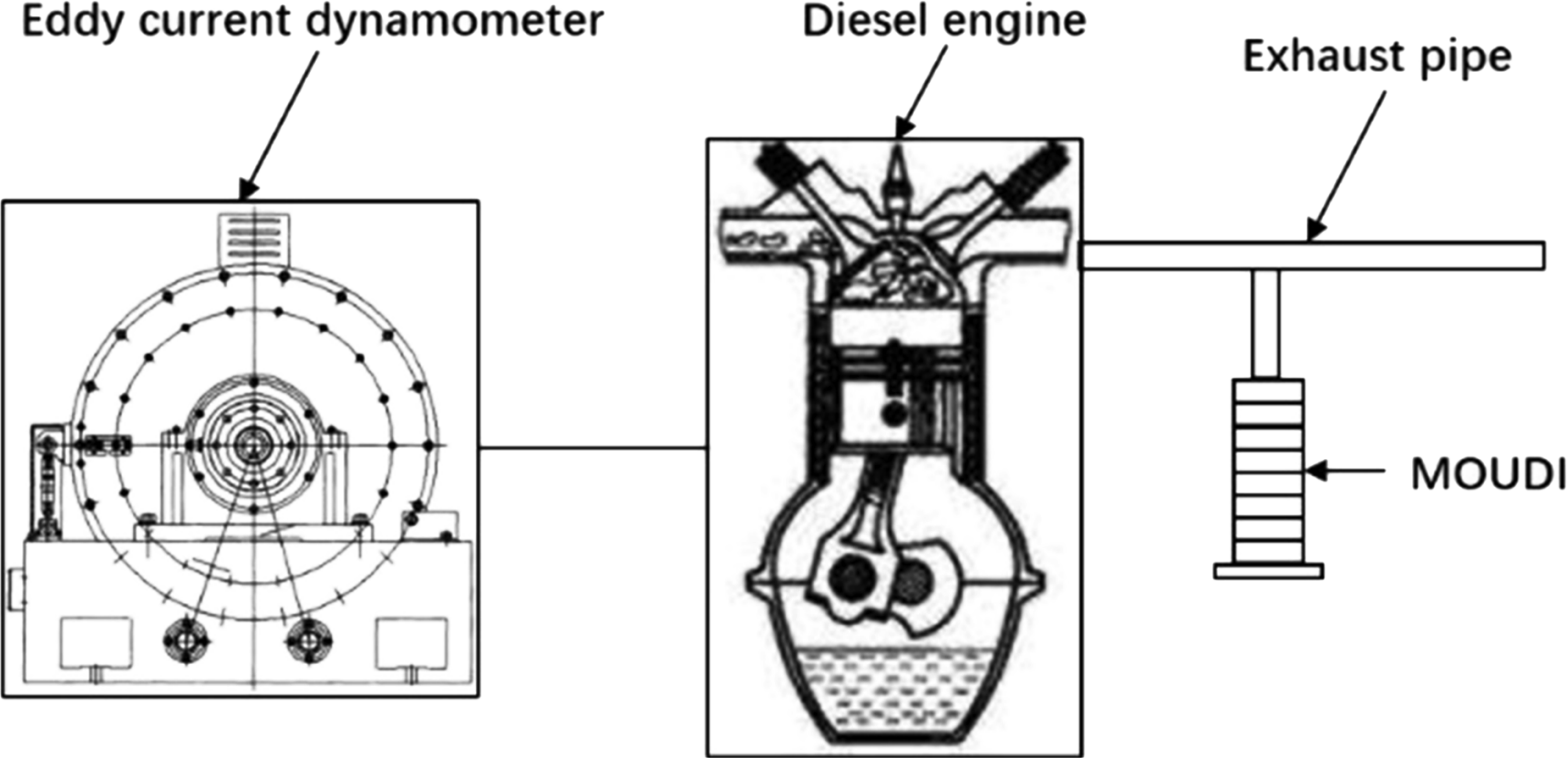
Schematic of the diesel particulate collection test.

Schematic of the diesel particulate collection test is displayed in Figure 1. The experiment was carried out on a diesel engine with a cylinder diameter of 86 mm. Main performance parameters of the diesel engine are listed in Table 3. The experiment was carried out on a stable diesel engine without adjusting the injection timing, running under the conditions of 2000 rpm and 75% load. During the test, the lubricating oil temperature of the diesel engine was maintained at 85 + 5 °C. The MOUDI was used to collect PM of BM10, BM200, and B100. The details of the MOUDI are displayed in Table 4. The position of the MOUDI was about 5 times that of the exhaust pipe diameter. According to the diffusion process of exhaust gas in the actual environment, in order to reduce the inlet temperature of the MOUDI, the collection system was cooled by air. During the sampling, standard aluminum foil filter paper (47 nm, MSP) was used to collect PM samples. The sampling flow was 30 L/min, and the sampling time was 40 min. Particulate samples for the thermogravimetric test were selected as 2 mg. In order to avoid the influence of environmental factors on particulate composition, the collected particulates were sealed and stored in containers. In order to reduce the measurement error, three measurements were carried out under the same working condition to calculate the average values.

**Table 3:** Engine Specifications

type	direct-injected, 4 stokes, air-cooled, natural aspiration
number of cylinders	1
cylinder bore (mm) × stroke (mm)	86 × 70
compression ratio	19
rated power (kW)/speed (rpm)	5.7/3000
nozzle number × orifice diameter (mm)	4 × 0.24
injection advanced angle (°CA BTDC)	12

**Table 4:** Particulate Collection and Analysis Equipment

equipment	model	manufacturer
micro-orifice uniform deposition impactor	MOUDI	MSP, USA
SEM	JSM-7001F	JEOL, Japan
thermogravimetric	TGA/DSC1	Mettler, Switzerland

#### 2.2.2 SEM

Particulates of B100, BM10, and BM20 were collected at an atmospheric environment. The micromorphology of the particulate was investigated using a JSM-7001F thermal field emission SEM. The details of the SEM test are displayed in Table 4. The electron beam was 1 pA–200 nA under a thermal field electron microscope, the acceleration voltage was 0.5–30 kV, the resolution was 1.2 nm(30 kV)/3.0 nm(1 kV), and the magnification was 10–500,000 times. Before the test, six kinds of particulate samples were treated by drying, sticking, and plating. Then, the suitable acceleration voltage, condenser current, working distance, objective diaphragm, and scanning speed were selected to ensure that the image can satisfy the requirement of the study. In the experiment, the magnification of the SEM was determined to be 50,000 times.

#### 2.2.3 Thermogravimetric Analysis

Thermogravimetric analysis was used to test the quality of the sample in the heating furnace with time and temperature. The experiment was carried out on a TGA/DSC1 thermogravimetric analyzer produced by Mettler-Toledo in Switzerland. The details of the thermogravimetric test are displayed in Table 4: temperature range: room temperature to 1600 °C; temperature accuracy: ±0.3 °C; heating rate: 0.1–150 °C/min; sample load: 0–1000 mg; balance sensitivity: 0.1 μg; temperature resolution: 0.0001 °C. The particle sample was weighed using the MX5-type microelectronic balance of Mettler-Toledo Company, Switzerland. The combustion temperature ranges of volatile matter and soot were different for diesel engine combustion particulates. Therefore, through the thermogravimetric test, volatile substances and soot can be distinguished. During the experiment, the oxidation gas was O_2_ and the protective gas was N_2_. The oxidation gas and the protective gas flow rate was 50 L/min, the heating rate was selected at 15 K/min, the temperature interval was 20–750 K, and the initial mass of the PM was about 2 mg.

The Coats–Redfern method^17^ was used to analyze the kinetic characteristics of pyrolysis of particulate samples in the O_2_ atmosphere. The process of calculating the activation energy of diesel particulate pyrolysis by the Coats–Redfern method was as follows:
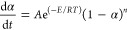
1where α is the mass change rate, *A* is the pre-exponential factor, *T* is the reaction temperature, *E* is the activation energy, *n* is the reaction order (diesel particulate oxidation, *n* = 1), *R* is the ideal gas constant, β is the heating rate, and β = d*T*/d*t* = 20 °C/min.

Then, eq 1 can be written as:
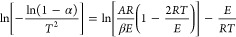
2

Normally, *E* ≫ *RT* and 1 – (2*RT*/*E*) ≈ 1. Thus, ln(*AR*/β*E*(1 – 2*RT*/*E*)) can be approximated as a constant. Equation 2 can be simplified as follows:
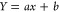
3

According to the linear fitting method, the slope of a straight line was −*E*/*R*, and the intercept of a straight line was ln(*AR*/β*E*). Thus, the pre-exponential factor *A* and the activation energy *E* can be calculated.

## 3 Results and Discussion

### 3.1 Microscopic Morphology of Particulates

**Figure 2:**
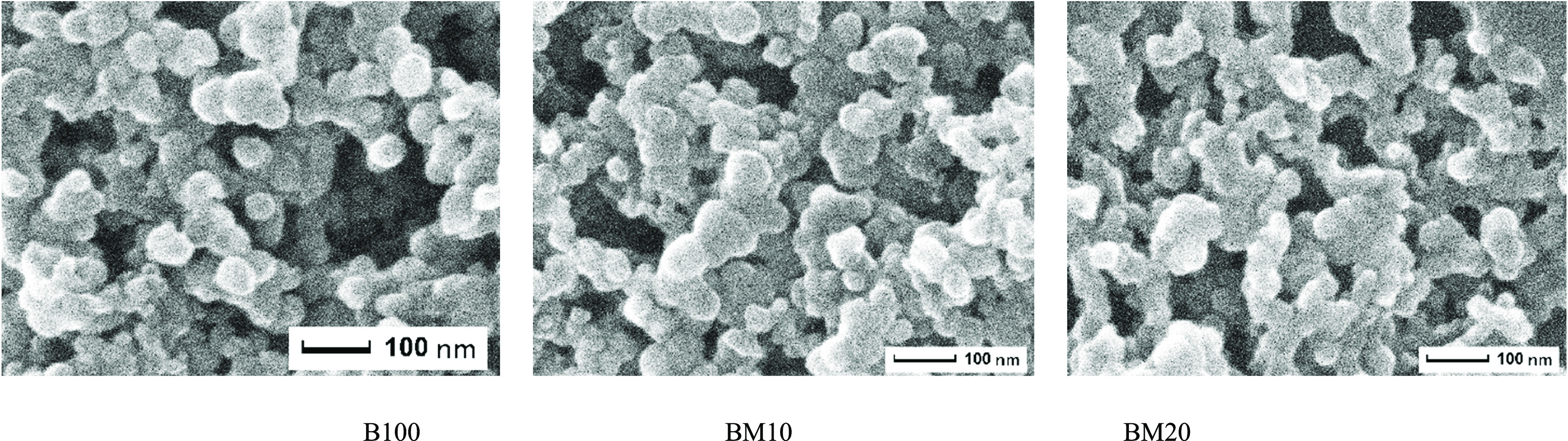
SEM images of B100, BM10, and BM20 particulates.

SEM images with 130,000 times enlargement of particulates produced by diesel engines fueled with B100, BM10, and BM20 are shown in Figure 2.

It can be seen that the particulates were mainly formed by the accumulation of quasi-spherical basic carbon particles with different particle sizes, forming clusters of particles with different densities. The main component of biodiesel was fatty acid methyl ester, with the functional group structure of −OCH_3_ and −(C=O)–. The connection between the carbon atom and oxygen atom was composed of a C–O single bond and a C=O double bond, and the C=O bond needed to absorb higher energy to be broken down to produce active oxygen free radicals (−O) at high temperature.^18^ At the beginning of the combustion reaction, the oxidation cracking process took longer time and the chemical reaction rate decreased, which resulted in the inadequate combustion of the fuel in the cylinder and more unburned soluble organic matter adhering on the surface of the particulates. As a result, the outline of particulates on the SEM images became blurred, the viscous force between particulates increased, and a large number of basic carbon particles accumulated.

To calculate the particle size on the SEM images, the software of Nano Measure was used. The basic principle of particle size calculation was to count the diameter of particulates per unit area and calculate the average value. Therefore, the average diameters of B100, BM10, and BM20 were calculated and they were 35, 32.6, and 31.2 nm, respectively. With the increase of methanol blending ratio, the particle size of the blend was reduced. On the one hand, methanol had the characteristics of a low cetane number and high latent heat of vaporization. With the increase of methanol content in the blend, the heat required for methanol gasification was increased, which resulted in relatively low temperature before the combustion in the cylinder. Thus, the ignition delay period was prolonged and the start time of combustion was backward. Then, more fuel was burned in the pre-mixed combustion stage and less fuel was burned in the diffusive combustion stage, resulting in less dry soot produced. Therefore, the formation of large particle size particulates was inhibited. On the other hand, the high temperature pyrolysis of methanol produced a large number of active free radicals, such as −O and −OH, which can oxidize the intermediate products (such as ethylene and acetylene) and produce CO_2_, H_2_, etc. The formation paths of monocyclic and polycyclic aromatic hydrocarbons were reduced, and the formation of dry soot was inhibited. Thus, the nucleation probability of particulates reduced and the particle size decreased gradually.

### 3.2 Volatility of Particulates

Adding methanol to biodiesel can change the composition and activity of particulates. In this paper, the pyrolysis test of methanol/biodiesel combustion particulates was carried out to analyze the volatilization and oxidation of particulates. SOF in diesel engine particulates was mainly composed of branched alkanes and *n*-alkanes, and its content was greatly affected by combustion temperature in the diesel engine.^19^ When the combustion temperature was lower, the dry soot finds it easy to adsorb and agglomerate more SOF components. References (20) and (21) showed that the main source of SOF in the methanol combustion process was pyrolysis of unburned esters. Because SOF was sensitive to temperature change, the volatilization characteristics of SOF and other volatiles in the particulates were investigated by temperature control in a N_2_ atmosphere.

**Figure 3:**
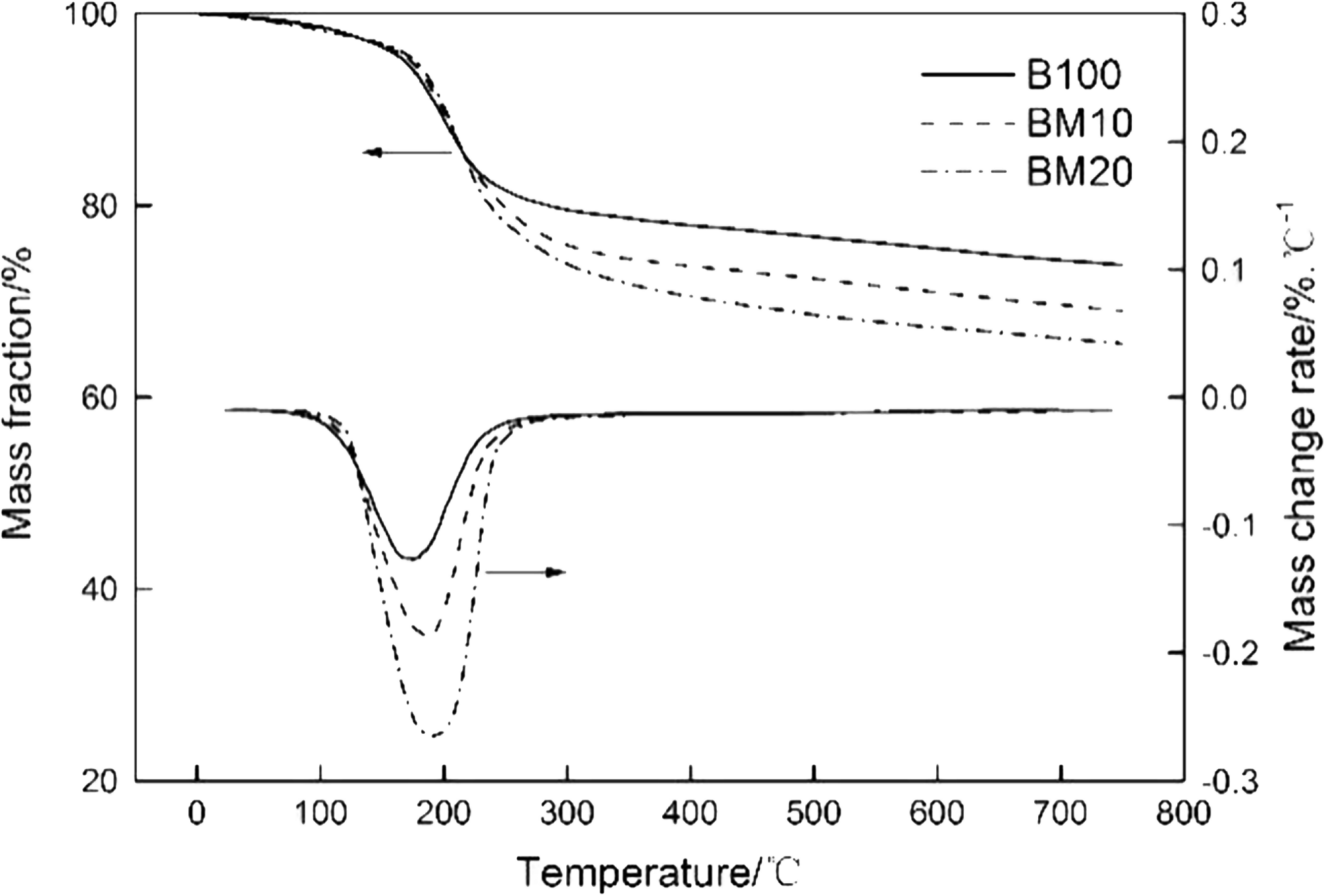
TG and DTG curves of particles in a N_2_ atmosphere.

Thermogravimetric tests were carried out on the three sampling results of each diesel engine working condition, and the average value was taken. The results of the thermogravimetric tests show that the error of the three measurements of particulates is less than 3%, which indicates that the repeatability of the test is good. Figure 3 shows the TG-DTG curves of exhaust particulates of B100, BM10, and BM20 in a N_2_ atmosphere. The TG curve showed that the mass of particulates was reduced with the increase of reaction temperature, and the absolute value of the corresponding DTG curve reflected the change rate of mass in the process of particulate reaction. According to the TG curve, the first stage was the evaporation process of water in the particulates in the temperature range of 40–110 °C. According to the data in Table 5, the mass change of H_2_O in the particulates was about 2.6 to 3.5%. This indicated that the water content in the particulates was increased with the increase of methanol mixing ratio. The second stage was SOF volatilization. The temperature range was 121–300 °C. From the DTG curve and test data, it can be seen that the exhaust particulates of B100, BM10, and BM20 showed obvious mass change rate peaks at about 200 °C. In addition, with the increase of methanol mixing ratio, the corresponding peak temperature increased and SOF content increased from 19.2 to 24.7%. This was mainly due to the lower heat value and the higher latent heat of vaporization of methanol. With the increase of methanol blending ratio in biodiesel, the heat value of the blend was reduced and the latent heat of vaporization was increased. During the combustion process, the maximum combustion temperature and thermal efficiency were reduced. Then, the unburned esters were increased and the organic components produced by pyrolysis were increased, resulting in the increase of SOF in exhaust particulates. According to the explanation of particle size, the addition of methanol in biodiesel was beneficial to the complete combustion of the fuel, and in the high-temperature pyrolysis, methanol produced a large number of active free radicals, resulting in less dry soot produced. Thus, with the addition of methanol, the soot content in particulates was reduced. The third stage was 400–750 °C, which indicated the mass change of soot and other substances in a N_2_ atmosphere. With the increase of temperature, there was no obvious mass change on the TG curve. It showed that the volatile matter in the particulates had been completely heated and precipitated. The mass change rate of the DTG curve was generally lower than that of the SOF volatilization stage and tended to be zero, indicating that the quality of soot had little change in the N_2_ atmosphere at this stage.

**Table 5:** Contents of Each Component of the Particle Sample

blends	H_2_O (%)	SOF (%)	soot (%)
B100	1.3	19.2	79.5
BM10	1.5	22.6	75.9
BM20	1.8	24.7	73.5

### 3.3 Oxidation Characteristics of Particulates

**Figure 4:**
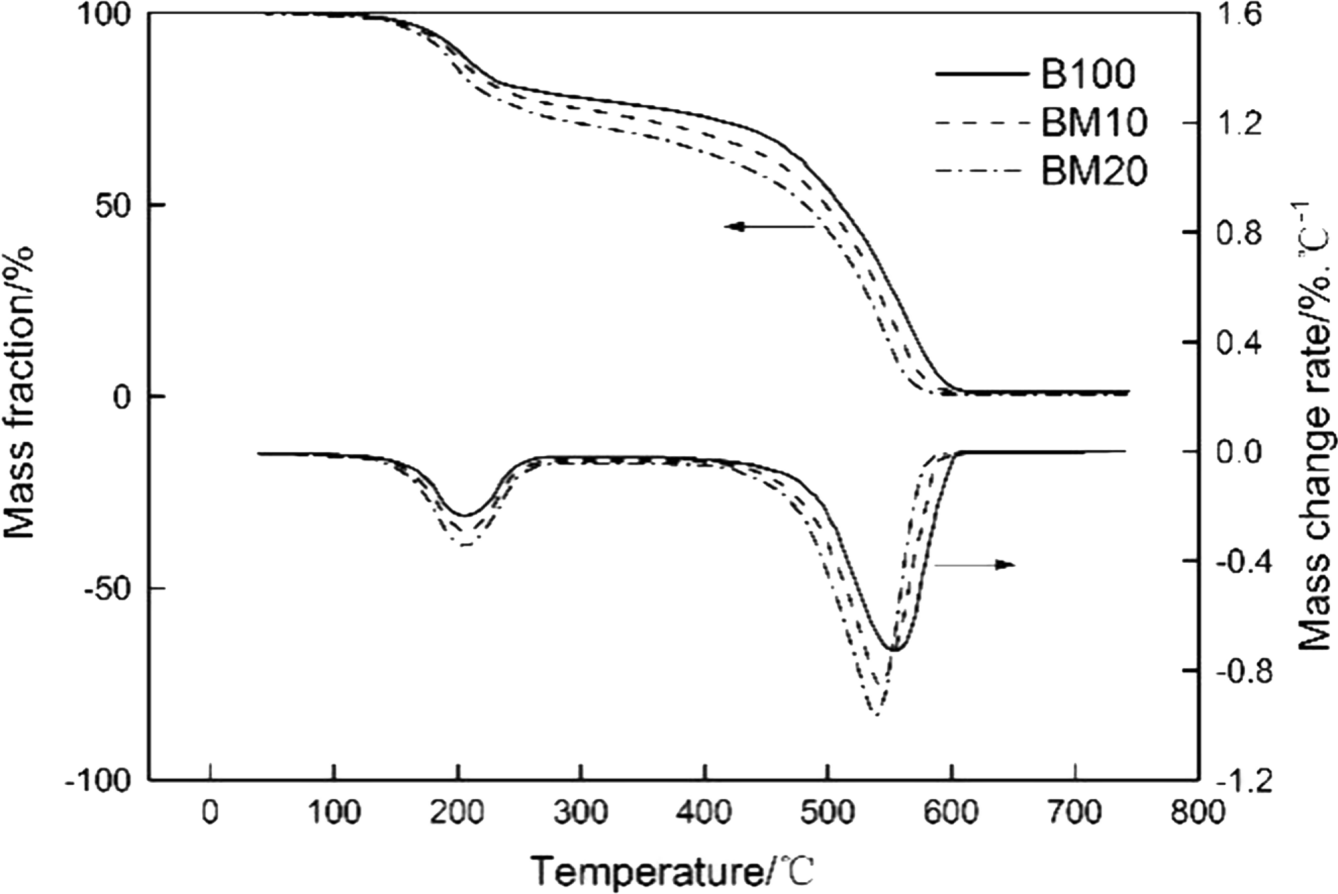
TG and DTG curves of particles in an O_2_ atmosphere.

Pyrolysis was the thermochemical conversion process of material thermal decomposition, which can occur in the initial or associated reactions of the gasification and combustion process. Figure 4 shows the TG/DTG curves of B100, BM10, and BM20 exhaust particulates in the O_2_ atmosphere. According to the TG curve, the heating process of particulates were mainly divided into three reaction stages, namely, water evaporation stage (stage 1: 40–110 °C), SOF volatilization stage (stage 2: 121–300 °C), soot oxidation stage (stage 3: 418–635 °C). There were two peaks of mass change rate in the DTG curve. The first mass change rate peak appeared at about 200 °C, which characterized the pyrolysis characteristics of SOF components in the O_2_ atmosphere. Compared with the N_2_ atmosphere, the mass change rate of SOF components increased significantly, showing the increase of the absolute peak mass change rate. This was because SOF volatilization occurred simultaneously with the oxidation reaction during the heating process in the O_2_ atmosphere, and the rate of mass change was increased. The second mass change rate peak occurred at about 600 °C, which characterized the oxidation process of soot components. The soot component accounted for 79.5, 75.9, and 73.5% of the total mass of exhaust particulates from B100, BM10, and BM20. The peak rates of absolute mass change were 0.73, 0.86, and 0.97%/°C, and the corresponding peak temperatures were 551, 542, and 538 °C, respectively.

It can be seen that with the increase of methanol mixing ratio, the carbon mass in particulates was reduced, the mass change rate was increased, and the peak temperature moved forward. This was mainly due to the increase of oxygen content in the blend fuels by mixing methanol, and more oxygen-containing groups were adsorbed on the surface of particulates after combustion, so the oxidation combustion rate of SOF components was faster. Increasing the oxygen content also improved the combustion hypoxia. More OH free radicals were produced in the initial stage of combustion, which can reduce PAHs and their precursors by the oxidation of free radicals, inhibiting the growth of soot particulates. At the same time, there was no C–C bond in methanol, and the formation of soot precursors, such as C_2_H_2_ and C_3_H_3_, were further reduced, and the emission of particulates was reduced. After mixing methanol, the oxygen content of fuel was increased, and the C–H bond, which was far from the oxygen group in particulate carbon, would be broken and more organic carbon was formed. Compared with elemental carbon, organic carbon had better oxidation characteristics.^22,23^ Therefore, when the temperature rose to a certain level, organic carbon burned rapidly, resulting in an increase in the rate of soot mass reduction.

### 3.4 Characteristic Temperature

The characteristic temperature is a parameter to characterize the volatilization and combustion characteristics of particulates. In this paper, four characteristic temperatures were selected to analyze the volatilization and combustion characteristics of particulates: SOF precipitation temperature *T*_SOF1_; initial combustion temperature *T*_SOF2_ and ignition temperature of soot components *T*_soot_; burnout temperature of soot components *T*_end_; maximum mass change rate *Max1* and corresponding temperature of SOF *T_Max1_* in a N_2_ atmosphere; maximum mass change rate *Max2* and corresponding temperature of SOF *T_Max2_* in an O_2_ atmosphere; and maximum mass change rate *Max3* and corresponding temperature of soot *T_Max3_* in an O_2_ atmosphere. Table 6 shows the characteristic temperatures of B100, BM5, BM10, and BM15 exhaust particulates. The oxygen content of methanol was higher than that of biodiesel.

**Table 6:** Characteristics Temperature of Particulates^a^

parameters	*T*_SOF1_ (°C)	*T*_SOF2_ (°C)	*T*_soot_ (°C)	*T*_end_ (°C)	*Max1* (%·°C^–1^)	*T_max1_* (°C)	*Max2* (%·°C^–1^)	*T_max2_* (°C)	*Max3* (%·°C^–1^)	*T_max3_* (°C)
B100	172	170	488	590	–0.13	177	–0.24	206	–0.73	555
BM10	177	161	466	578	–0.19	183	–0.30	206	–0.86	542
BM20	180	155	448	564	–0.27	188	–0.35	206	–0.97	538

a Note: *T*_SOF1_ and *T*_SOF2_ were the precipitation temperature and the initial combustion temperature of SOF respectively; *T*_soot_ and *T*_end_ were the ignition temperature and the burnout temperature of the soot component, respectively.

As can be seen from Table 3, with the increase of methanol content, the precipitation temperature *T*_SOF1_ changed slightly in a N_2_ atmosphere, rising from 172 °C of B100 to 180 °C of BM20. Compared with B100, the *T*_SOF2_ of exhaust particulates blended with methanol was reduced slightly compared with B100, but the *T*_SOF2_ of BM10 and BM20 particulates was reduced slightly. *T*_soot_ and *T*_end_ of particulates were reduced with the increase of methanol mixing ratio, and *T*_end_ ranged within 590–564 °C. This was mainly due to the increase of oxygen content in the blend and the adsorption of more oxygen-containing organic compounds in the particulates. When the temperature rose to a certain level, the soluble organic components on the surface of the particulates precipitated and oxidized rapidly, which resulted in the decrease of *T*_SOF2_. With the increase of methanol mixing ratio, the particulate disorder and looseness of the blend fuel were increased, and heat transfer resistance was reduced. Therefore, in the pyrolysis process, the particulates can ignite at a lower temperature, which showed the smaller *T*_soot_. The absolute value of *Max1*, *Max2*, and *Max3* for BM10 and BM20 were higher than B100, and *T_Max1_*, *T_Max2_*, and *T_Max3_* for BM10 and BM20 were lower than B100. This was because the carbon chain length of methanol was shorter than that of biodiesel. Thus, the carbon chain length of the organics produced after methanol combustion was also shorter than biodiesel. From the perspective of material properties, shorter chain organics were easier to volatilize in the N_2_ atmosphere and easier to oxidize in the O_2_ atmosphere.

### 3.5 Kinetic Parameters of Pyrolysis

The activation energy parameters of particulates were obtained, and the pyrolysis difficulty degree of particulates produced from the blend by mixed with methanol was judged. According to the TG/DTG curve, the activation energy of particulate pyrolysis reaction parameter *E* (activation energy) was obtained by the integral method. The activation energy is represented by the height of the barrier (energy barrier). The larger the activation energy, the more energy is needed for particulate pyrolysis. Table 7 lists the fitting equations and parameters of pyrolysis kinetics for particulates of B100, BM10, and BM20. The results showed that the fitting linear coefficients were all above 0.98, which indicated that the fitting accuracy was high and the activation energy of particulates was reduced with the increase of methanol mixing ratio. This was because with the increase of methanol blending ratio, the organic carbon content in the particulate was increased. In addition, the disorder degree of the particulate was increased, and the graphitization degree of particulate carbon was reduced, which leads to more easily oxidized particulates. Therefore, with the increase of methanol mixing ratio, the activation energy of the particulate pyrolysis reaction was reduced and the energy required for particle pyrolysis was reduced.

**Table 7:** Kinetic Parameters of Particulate Pyrolysis

fuels	fitting equations	*R*^2^	*E* (kJ·mol^–1^)
B100	*y* = −16.8*x* + 4.86	0.9952	139.7
BM10	*y* = −15.9*x* + 4.13	0.9964	132.2
BM20	*y* = −15.1*x* + 3.76	0.9948	125.5

## 4 Conclusions

In this paper, the particulates of methanol/biodiesel blends with methanol mixing ratios of 0, 10, and 20% were collected under the experimental conditions of 2000 rpm and 75% load. The effects of methanol blending on the structure parameters and oxidation characteristics of methanol/biodiesel particulates were analyzed by means of SEMS and TG experiments. The main conclusions were as follows:

(1) With the increase of methanol blending ratio in biodiesel, the particulate surface of methanol/biodiesel was adhered to unburned SOF. Therefore, the H_2_O and SOF contents in particulates were increased, and soot content was reduced. The average diameters of B100, BM10, and BM20 were 35, 32.6, and 31.2 nm, respectively.
(2) With the increase of methanol mixing ratio, the precipitation temperature of SOF component *T*_SOF1_ was increased slightly, the initial combustion temperature *T*_SOF2_ was reduced, the initial combustion temperature of soot component *T*_soot_ was reduced, the burnout temperature of soot *T*_end_ was reduced, and the activation energy of the particulate pyrolysis reaction was also reduced. This indicated that the energy required for the particulate pyrolysis reaction was reduced and the total pyrolysis reaction time of particulates was shortened with the increase of methanol content. Therefore, the oxidative reaction of particulates was easier to carry out due to the addition of methanol.
(3) The addition of methanol to biodiesel can improve the oxidation characteristics of particulates. Further study on the process of methanol/biodiesel combustion from the perspective of chemical reaction kinetics is helpful to reveal the mechanism of methanol or alcohol fuel affecting the oxidation characteristics of particulates.
